# Supplementary material for: Detection of soil-transmitted helminths and Schistosoma spp. by nucleic acid amplification test: Results of the first 5 years of the only international external quality assessment scheme
Source: PLoS Negl Trop Dis. 2024 Aug 8;18(8):e0012404. doi: 10.1371/journal.pntd.0012404 (PMC11335160; doi:10.1371/journal.pntd.0012404)
Supplement: Attachment — Submitted filename: Schutte et al answers to reviewer comments 20240715.docx [file pntd.0012404.s003.docx]

**Responses to review comments**

Reviewer #1: Why was the scheme designed for only 1 challenge per year? How does this align with recipient requirements for PT frequency?

*Answer: Accreditation regulations for medical laboratories do not impose at which frequency proficiency testing should be performed. For molecular detection methods, such as real-time PCR, participation in an external quality assessment scheme that distributes samples once a year, is quite a common procedure. In addition, for logistical and economic reasons samples were distributed once per year. A higher frequency of distribution will increase the participation cost substantially, which would hamper participation of laboratories, especially in endemic countries. This rationale has been added to the text (lines 147-148 in the revised manuscript with track changes).*

*We have adjusted the text in the methodology section (lines 130-148) in the revised manuscript with changes), to emphasize that the design and organization of HEMQAS were described in great detail in a previous study ((Cools P, Vlaminck J, Albonico M, Ame S, Ayana M, Jose Antonio BP, et al. Diagnostic performance of a single and duplicate Kato-Katz, Mini-FLOTAC, FECPAKG2 and qPCR for the detection and quantification of soil-transmitted helminths in three endemic countries. PLoS Negl Trop Dis. 2019;13(8):e0007446).*

Reviewer #2: Line 131: Insufficient information is given about the samples distributed. How were they "Well validated" and by whom? What were the egg counts? Were the stools fresh or frozen? Were they sent out "dry" or in PCR extraction buffer, for example? How were the DNA samples sent out? Purified DNA or DNA seeded into negative stools? What was the quantity of DNA sent out? Was the same quantity sent out each time?

*Answer: We have adjusted the methods section to provide additional information regarding sample preparation and distribution (lines 131 – 148 in the revised manuscript with track changes). As explained above, a very detailed description of the distribution and validation can be found in the reference to the pilot study (Cools P, Vlaminck J, Albonico M, Ame S, Ayana M, Jose Antonio BP, et al. Diagnostic performance of a single and duplicate Kato-Katz, Mini-FLOTAC, FECPAKG2 and qPCR for the detection and quantification of soil-transmitted helminths in three endemic countries. PLoS Negl Trop Dis. 2019;13(8):e0007446).*

Reviewer #2: Line 190: Were all the stools positive by light microscopy? if so, PCR performance was poor.

*Answer: Although for most stool samples the presence of helminth eggs and/or larvae was confirmed by light microscopy in the original stool specimen from which the distributed samples were prepared, it was not part of the standard procedure nor a prerequisite for inclusion of a clinical specimen (information added to the revised manuscript in lines 142-148). The presence or absence of the target helminths in the distributed samples (which were diluted during the preparation process) was certified only by PCR by the expert laboratories (see Cools et al 2019, for details). The concentration of helminth eggs and/or larvae was often below microscopic detection limits.*

Reviewer #2: Discussion line 298: Please rephrase this line; should read positive for....

*Answer: This writing error has been corrected (see line 313 in the revised manuscript)*

Acknowledgements: Those named as giving expert technical assistance will have done a substantial amount of work. Please consider whether they meet the criteria for full authorship.

*Answer: The organization of the EQAS for molecular detection of helminths in stool was indeed a considerable amount of work, not only for the expert laboratories but especially also for the coordinating and material preparation laboratory. For a careful and proper balance in authors, a single author from each expert laboratory could be included and each expert laboratory suggested the most appropriate researcher for this authorship.*

Reviewer #2: Minor grammatical edits are needed.

*Answer: Multiple grammatical corrections have been made after another check.*

**Responses to editorial comments**

1) Thank you for stating" The participants were asked to report the detected helminths species with a corresponding Cq136 . Concurrently, all participating laboratories received an online questionnaire in which they were asked to specify the DNA extraction and NAAT protocol that was used for their NAAT.

Please include:

i) The full name(s) of the Institutional Review Board(s) or Ethics Committee(s)

ii) The approval number(s), or a statement that approval was granted by the named board(s)

iii) A statement that formal consent was obtained (must state whether verbal/written) OR the reason consent was not obtained (e.g. anonymity). NOTE: If child participants, the statement must declare that formal consent was obtained from the parent/guardian.].

*Answer: In this study participants are laboratories that participated in an external quality assessment scheme. Their results and answers to additional question have used with consent to examine the variation in reported results in order to improve the quality of this relatively novel laboratory test. So, no patients, nor patient information was involved, and thus nu ethical approval is required.*

*To prevent this confusion, “participant” has often been changed into “participating laboratories”.*

2) Please ensure that the CRediT author contributions listed for every co-author are completed accurately and in full.

At this stage, the following Authors/Authors require contributions: Annemiek H.J. Schutte, Rob Koelewijn, Sitara S.R. Ajjampur, Bruno Levecke, James S. McCarthy, Rojelio Mejia, Steven A. Williams, Jaco J. Verweij, Lisette van Lieshout, and Jaap J van Hellemond. Please ensure that the full contributions of each author are acknowledged in the "Add/Edit/Remove Authors" section of our submission form.

*Answer: Author-contribution information has been added to the main text of the document.*

3) We do not publish any copyright or trademark symbols that usually accompany proprietary names, eg ©, ®, or TM (e.g. next to drug or reagent names). Therefore please remove all instances of trademark/copyright symbols throughout the text, including: 2016© and Graphpad Prism 9.1.4©, On line no 173, and 174.

*Answer: Both copyright signs have been deleted.*

4) Please upload all main figures as separate Figure files in .tif or .eps format.

*Answer: Figures files have been uploaded in TIF format.*

5) Tables should not be uploaded as individual files. Please remove these files and include the Tables in your manuscript file as editable, cell-based objects.

*Answer: Table 1 has been uploaded as separate cell-base object file.*

6) We note that your Data Availability Statement is currently as follows: [All data is described in the manuscript. No supplementary data required.]

Please confirm at this time whether or not your submission contains all raw data required to replicate the results of your study. Authors must share the “minimal data set” for their submission. PLOS defines the minimal data set to consist of the data required to replicate all study findings reported in the article, as well as related metadata and methods.

For example, authors should submit the following data:

- The values behind the means, standard deviations and other measures reported;

- The values used to build graphs;

- The points extracted from images for analysis.

Authors do not need to submit their entire data set if only a portion of the data was used in the reported study.

If your submission does not contain these data, please either upload them as Supporting Information files or deposit them to a stable, public repository and provide us with the relevant URLs, DOIs, or accession numbers.

*Answer: The data from which the figures was prepared has been added as supporting information documents 1 and 2.*
